# Diagnosis, assessment, and treatment of childhood eczema in primary care: cross-sectional study

**DOI:** 10.3399/bjgpopen17X100821

**Published:** 2017-05-03

**Authors:** Laureen Jacquet, Daisy M Gaunt, Kirsty Garfield, Matthew J Ridd

**Affiliations:** 1 Medical Student, School of Social and Community Medicine, University of Bristol, Bristol, UK; 2 Research Associate in Medical Statistics, School of Social and Community Medicine, University of Bristol, Bristol, UK; 3 Research Associate in Medical Statistics, Bristol Randomised Trials Collaboration, School of Social and Community Science, University of Bristol, Bristol, UK; 4 Research Associate in Health Economics, School of Social and Community Medicine, University of Bristol, Bristol, UK; 5 Research Associate in Health Economics, Bristol Randomised Trials Collaboration, School of Social and Community Medicine, University of Bristol, Bristol, UK; 6 Consultant Senior Lecturer in Primary Health Care, School of Social and Community Medicine, University of Bristol, Bristol, UK

**Keywords:** atopic dermatitis, eczema, diagnostic criteria, disease management, treatment

## Abstract

**Background:**

The majority of children with eczema in the UK are looked after in primary care yet we know little about their care in this setting.

**Aim:**

To compare the diagnosis, assessment, and treatment of eczema in primary care with published diagnostic criteria and management guidelines.

**Design & setting:**

Cross-sectional study using data from a randomised controlled feasibility study. General practices, UK.

**Method:**

Baseline data from children aged 1 month to 5 years recruited ‘in-consultation’ for the Choice of Moisturiser in Eczema Treatment (COMET) feasibility study was used. These included clinician diagnosis and global severity assessment; the parent-completed Patient Orientated Eczema Measure (POEM); a questionnaire about eczema treatments, including use of topical corticosteroid (TCS); and, the Eczema Area Severity Index (EASI) carried out by trained researchers. Descriptive analyses were undertaken to compare diagnoses with UK diagnostic criteria, severity assessments, and treatment with the National Institute for Health and Care Excellent (NICE) guidance.

**Results:**

Data were available for 90 participants. Only 46% of participants labelled as having eczema met the UK diagnostic criteria. Agreement between the global severity assessment by a healthcare practitioner with the EASI and POEM measures of eczema severity were 44% and 48% respectively. Emollients and TCSs were underused with 44% of participants not using any emollient and 46% using one or more TCSs. The ‘match’ between eczema severity and TCSs potency was poor.

**Conclusion:**

Discrepancies were found between the diagnosis, assessment, and treatment of children with eczema in primary care, and UK diagnostic criteria and guidelines. Further investigation to explore the reasons for this discordance, and whether it matters, is needed.

## How this fits in

Most children with eczema are diagnosed and managed in primary care but little is known about their assessment or treatment. It was found that less than half of children diagnosed with eczema in primary care met the UK atopic eczema diagnostic criteria. Agreement between clinician, parent, and ‘objective’ assessments of severity was also limited. Reported non-use of an emollient was common and there was often a discordance between the topical corticosteroid (TCS) potency and eczema severity.

## Introduction

Eczema (also known as atopic eczema/dermatitis) is one of the most common childhood disorders with high prevalence (0.2–24.6%) worldwide.^1^ The burden of the disease falls on pre-school aged children although it persists in a significant proportion through to adulthood.^2^ It is characterised by dry and itchy skin and can have a significant impact on the quality of life of the patient and their family.^3,4^

In countries like the UK, the majority of children with eczema are managed exclusively by GPs^5^, yet little is known about their diagnosis, assessment, or treatment in this setting. Emerson *et al* ^6^ found that of children with eczema in primary care, the majority (84%) had mild eczema, with 14% moderate, and 2% severe. However, this study is now dated (1998), and assessments were based on a single dermatologist’s clinical opinion, and no patient-reported measures were collected.

Treatment guidelines recommend the use of emollients as a leave-on treatment (250–500g weekly) and as soap substitutes, with different types for different purposes.^7^ For eczema 'flares', NICE advocates the use of TCSs with a potency that matches eczema severity, such as mild TCSs for mild eczema, or moderate TCSs for moderate eczema.^7^ Prescribing data in Scotland from 2000–2001 indicated that emollients are underprescribed and TCSs are used too much and at an inappropriate potency.^8^

Using data from the COMET feasibility study,^9^ the authors sought to better understand how eczema is currently diagnosed, assessed, and treated in primary care in the UK.

## Method

### The COMET study

Full details of the COMET study can be found in the protocol.^9^ The aim of the study was to determine the feasibility of recruiting young children with eczema and randomising them to one of four commonly used leave-on emollients. To be eligible to take part, children had to be between 1 month and 5 years of age, have a clinical diagnosis of eczema, and not known to be sensitive or allergic to any of the study emollients or their constituents.

Participants were recruited via 22 GP surgeries located in the West of England. All practices sent letters to potentially eligible children asking them to contact the study team if they were interested in taking part (the ‘self-referral’ pathway). The focus of this study is on participants who were recruited by clinicians, GPs, practice nurses (PNs), or practice pharmacists (PPs) in the 16 practices who recruited participants ‘in-consultation’.

### Data collection

As part of the consultation in which patients were consented and randomised to the study, the recruiting healthcare provider (HCP) made a global clinical assessment of eczema severity (clear, mild, moderate, and severe) in accordance with NICE guidelines,^7^ (Box 1) and the parent or carer was asked to complete POEM.^10^ A researcher subsequently collected data on the diagnosis as per the UK atopic eczema diagnostic criteria (Supplementary Box 1)^11^ and eczema severity assessed using EASI.^12^ Patient-reported eczema treatments, such as emollients, bath emollients, TCSs, topical calcineurin inhibitors (TCI) were collected using parent or carer self-report.

**Box 1.** Eczema severity assessment (taken from the NICE guideline CG57)^7^

**Clear:** normal skin, no evidence of active atopic eczema. **Mild:** areas of dry skin, infrequent itching (with or without small areas of redness). **Moderate:** areas of dry skin, frequent itching, redness (with or without excoriation and localised skin thickening). **Severe:** widespread areas of dry skin, incessant itching, redness (with or without excoriation, extensive skin thickening, bleeding, oozing, cracking and alteration of pigmentation).

### Data analysis

Analysis was done using Stata (version 13.1). POEM and EASI scores were calculated and categorised as per published guidance.^10,12^ The Index of Multiple Deprivation score (IMD) was calculated from the English IMD 2010 score (based on Lower Super Output Area) using the GeoConvert web application from the UK Data Service Census Support.^13^ Fisher’s exact test was used to examine differences between assessments of eczema severity by individual and groups of HCPs. Percentage agreement between different assessments of eczema severity and TCSs potency were calculated.

## Results

### Participant characteristics

**Figure 1.**
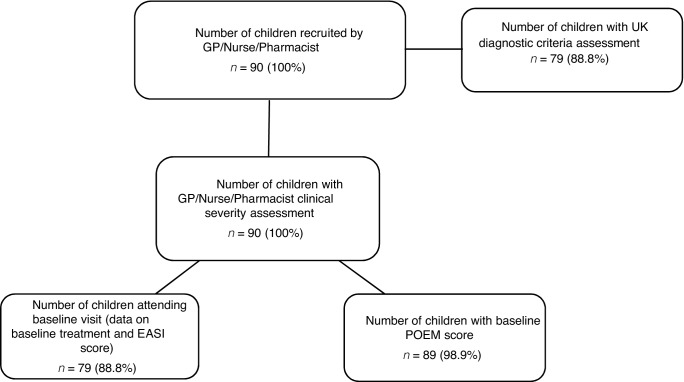
Participant flow.

Out of 197 participants, 90 were recruited 'in-consultation’ and were included in this analysis (Figure 1). The majority of patients recruited were of white-British ethnic origin (73%, Table 1). Other participant characteristics are summarised in Table 1.

**Table 1.** Participant characteristics

Demographics		*n*
Mean age, months (SD)	17.0 (12.6)	90
Females, *n* (%)	39 (43)	90
White, *n* (%)	57 (73)	78
Mean IMD score (SD)	27.6 (15.3)	88
**Eczema severity**		
Mean EASI (SD)	3.1 (3.4)	79
Mean POEM (SD)	10.3 (5.8)	89

SD = standard deviation. EASI = eczema area severity index. POEM = patient orientated eczema measure.

One parent or carer did not complete the POEM questionnaire, 10 participants did not attend their baseline visit, and baseline data were not collected on one participant. Therefore, data for POEM were available on 89 (99%); and UK diagnostic criteria and EASI on 79 (89%) of participants (Figure 1). Thirty-two per cent (25/79) had an eczema diagnosis of over 12 months duration.

### Eczema diagnosis and severity assessment

Forty-six per cent (36/79) of participants met the UK diagnostic criteria for atopic dermatitis. Clinical assessments of eczema severity were done by 26 different HCPs (21 GPs, 4 PNs, and 1 PP). One GP and one nurse each did 16 assessments (32/90 [36%] of total). Thirty-three per cent (26/80) of baseline (when EASI was collected) assessments took place within 5 days and 80% (64/80) within 10 days of referral (when the clinical and parent (POEM) outcome measures were collected).

HCPs assessed participants’ eczema as 4% clear, 61% mild, 31% moderate, and 3% severe (Table 2). There was no evidence of a difference in severity scoring between the types of HCP (Fisher’s exact test *P* = 0.736, Supplementary Table 1) or individual assessors (Fisher’s exact test *P* = 0.280).

**Table 2.** Eczema severity by method of assessment

	Number of children (%)		
Severity	Clinical	POEM	EASI
Clear	4 (4)	7 (8)	22 (28)
Mild	55 (61)	12 (24)	48 (61)
Moderate	28 (31)	50 (56)	8 (10)
Severe	33 (3)	11 (12)	1 (1)
Total	90 (100)	89 (100)	79 (100)

POEM = patient orientated eczema measure. EASI = eczema area severity index.

There was variation in the categorisation of eczema severity between clinical, POEM, and EASI (Table 2). Clinical and EASI assessments categorised 61% of participants as having ‘mild’ eczema, whereas according to the parent-reported measure POEM, more participants had ‘moderate’ (56%) eczema. Agreement between clinical assessment and EASI was 44% (35/79, Supplementary Table 2); and clinical assessment and POEM was 48% (43/89, Supplementary Table 3).

### Treatment: emollients and topical corticosteroids

Forty-four per cent (35/79) of said they were not using any emollient, while 41% (32/79) of the participants were only using one. Sixteen different brands were named (Supplementary Table 4). The majority (44/79, 56%) of participants were not using a bath emollient.

Forty-six per cent (36/79) of participants reported using one or more TCSs. Four reported using two different types of TCSs, and two of these were using two of mild potency. One participant was using three different types TCSs (two mild, and one potent). TCSs potency were as follows: 41% (32/79) mild, 4% (3/79) moderate, and 5% (4/79) potent TCSs. Nine different types of TCSs were used by respondersparticipants (Supplementary Table 5).

Only one participant out of 79 (1%) suffering from moderate eczema was using a TCI (tacrolimus monohydrate 0.03%, Protopic^®^, Astellas Pharma Tech Co., Ltd.).

### Eczema severity and topical corticosteroid potency

**Figure 2.**
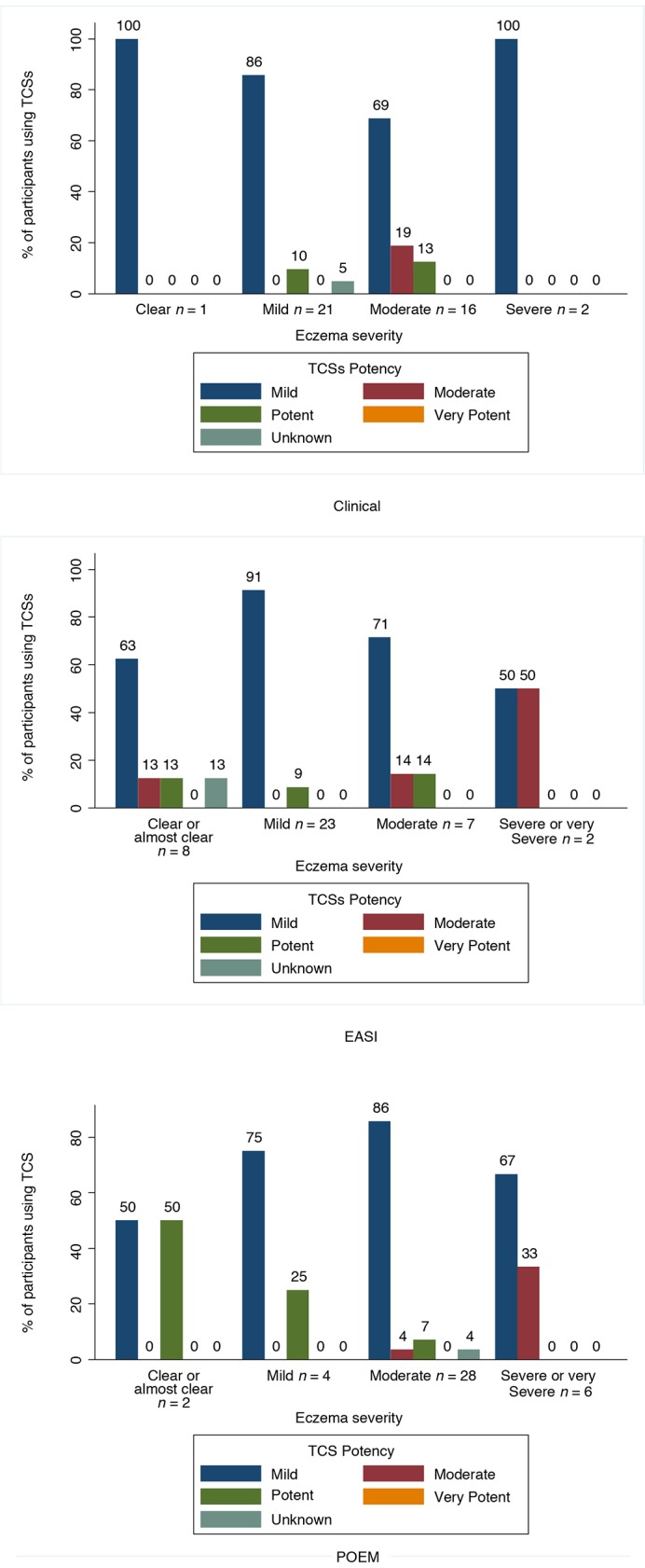
Topical corticosteroid use by categorical clinical, EASI, and POEM assessments of eczema severity. TCS = topical corticosteroid. EASI = Eczema Area Severity Index. POEM = Patient Orientated Eczema Measure.

Figure 2 compares TCSs potency use with clinical, EASI, and POEM assessments of eczema severity (Figure 2). Regarding the clinical eczema severity assessment, 86% (18/21) and 19% (3/16) of participants who reported using a potency of TCSs that ‘matched’ their ‘mild’ and ‘moderate’ eczema severity, respectively. None of the three participants with ‘severe’ eczema reported using a potent or very potent TCS.

With respect to eczema severity according to EASI, 91% (21/23) of ‘mild’ and 14% (1/7) of ‘moderate’ eczema sufferers reported using a mild and moderate potency TCSs, respectively. The one participant assessed as ‘severe’ eczema was not treated with a potent or very potent TCSs.

Regarding eczema severity based on POEM, 75% (3/4) participants with ‘mild’ eczema reported using a mild potency TCS. However, one participant considered as ‘clear’ or ‘almost clear’ reported use of a potent TCSs.

**Figure 3.**
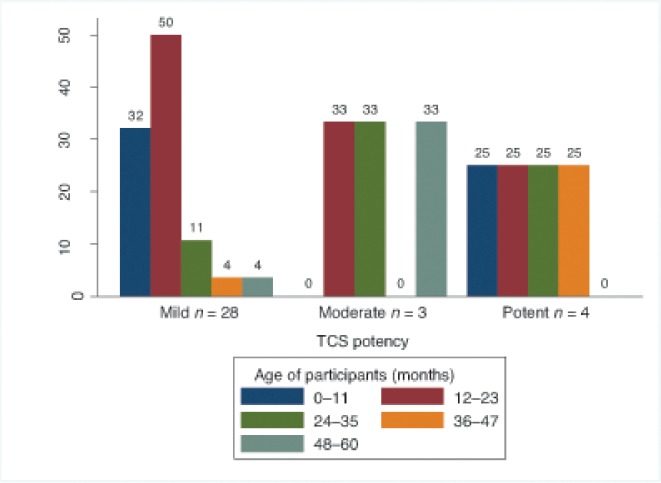
Topical corticosteroid potency by participant age.

It was hypothesised that reported use of TCSs by potency might be related to the age of the participants, especially for moderate and potent TCSs. As Figure 3 shows, age of the participantresponder, and the potency of the TCSs used did not appear to be associated.

## Discussion

### Summary

This is the first study to compare the diagnosis, assessment, and treatment of children with eczema in primary care with published diagnostic criteria and treatment guidance. Less than half (46%) of the participants recruited met the UK diagnostic criteria for atopic dermatitis. Agreement between clinical and categorised parent-reported (POEM) and observer 'objective' (EASI) assessments of severity was low (44% and 48%, respectively). A significant proportion of responders (44%) were not using any emollient, and there was poor concordance between eczema severity (according to clinical, EASI, and POEM) and reported potency of TCSs used. Children with ‘moderate’, ‘severe’ or ‘very severe’ eczema (categorised by any assessment measure) were most likely to report using a mild TCS.

### Strengths and limitations

Linked data on eczema diagnosis, severity (using three different methods) and treatment are presented for the first time. However, these data were collected as part of a feasibility study and thus comprise a modest number of participants who may not be representative of all children with eczema. Indeed, participants recruited into the study via the ‘in consultation’ pathway were younger and had higher baseline POEM scores, compared with those children recruited via the ‘self-referral’ pathway in the COMET study.^9^ Missing data, by virtue of incomplete questionnaires or failure to attend the baseline appointment, further reduced the number of respondersparticipants who could be inluded in some of the analyses. Clinical assessments were done by 26 different HCPs and no data on inter-rater reliability was available. While it is possible that HCPs assessments could have been influenced by parents/carers completing the POEM questionnaire in front of them, this seems unlikely, given the low level of agreement with EASI. One surgery recruited children to the study via the ‘in-consultation’ pathway particularly well, and as a consequence, the nurse and GP from this practice contributed 36% of the assessments, which included 50% of all nurse and 27% of all doctor assessments. Parent-reported use of medication may be unreliable and this was not compared against prescription data. Fluctuations in eczema severity in the time between the collection of the clinical and POEM assessments in the GP practice consultation and EASI at the baseline visit may explain some of the differences between these measures. Furthermore, the clinical and EASI assessments are based on clinical review of the evidence of eczema, whereas POEM asks the parent/carer about eczema symptoms (including itch and sleep disturbance) over the previous week.

### Comparison with existing literature

Eczema is a clinical diagnosis^13^ and the UK diagnostic criteria remain the most extensively validated instrument for defining atopic eczema and dermatitis.^14^ They perform well in children aged ≥3 years, but the diagnosis can be more difficult in infants.^15^ This may explain some of the discrepancy between the number of children with a clinical diagnosis of eczema and those meeting the UK diagnostic criteria for eczema.

There is no 'gold standard' for assessing eczema severity. POEM and EASI are validated patient-reported and 'objective' measures of eczema severity, recommended as core outcome measures in trials of eczema treatments.^16^ The discordance between clinical assessment and these two measures may be due to one of three reasons. First, the published categorisations of eczema severity for POEM and EASI could be wrong.^17–18^ This seems unlikely, as they have been derived using appropriate methods. Second, the different assessments are evaluating different aspects of childhood eczema. The clinical assessment is a global impression of severity, subject to individual clinician biases, and POEM is a measure of symptoms in the affected child over the previous week,^10^ whereas EASI relies purely on clinical signs at one point in time.^12^ Third, the categories of clinical assessment may be too crude. The NICE categories and descriptions were based on guideline group consensus, and are not underpinned by any research into their validity or utility.^7^

Published data regarding the use of treatments for eczema, with which to compare these findings, are limited. Santer and colleagues^8^ reported that emollients are underprescribed and that 15.9% of children were prescribed a potent or very potent TCSs. Adherence to topical treatments adherence declines over time,^19–20^ which may explain some of these findings. Much has been written about ‘TCSs phobia’,^21^ which may explain the low appropriate use of moderate or more potent TCSs among parents or carers, but this data could possibly also reflects underprescribing by primary care HCPs.^22^ Another reason for the apparent underuse of TCSs may be due to sensitivity of the treatment area, such as the face.^23^ Finally while the use of TCSs of a greater potency than disease severity by one category (for example, mild potency TCSs in children with clear or almost clear eczema), may be appropriate and reflect treatment ‘success’ (maintenance of disease remission), it is difficult to see how the reported use of potent or very potent TCSs in children with ‘clear’ or ‘almost clear’ eczema is justified.

### Implications for research and clinical practice

Studies of this type need replicating in larger populations with a wider range of clinicians and with further analyses of inter-rater reliability. Further work is also required to validate the NICE global assessment of eczema severity, ideally in relation to measures such as the POEM and EASI. Finally, research should be undertaken to explore the discrepancy between diagnostic criteria and treatment guidelines. This should not only seek to understand the reasons why, but also the consequences of, under- or over- treatment of eczema and short- and longer-term outcomes, including adverse effects.

In the meantime, primary care clinicians should heed concerns about diagnosis of eczema in infants, in particular distinguishing between eczema and seborrheic dermatitis.^24–25^ They should also confirm that patients with eczema prescribed a TCS are using an emollient alongside, and ensure that the potency of TCSs being used is appropriate for the eczema severity and site. They may want to consider routinely asking parents of children with eczema to complete the POEM.^26^ Unlike the NICE clinical global assessment categories of severity, POEM has been validated and provides information on the effect of the disease on the child, which cannot be determined from physical examination alone.

## Appendix 1

### Box S1. UK Atopic dermatitis diagnosis criteria

In order to qualify as a case of atopic eczema with the UK diagnostic criteria, the child:

Must have:

- History of itchy skin

Plus three or more of:

- Onset under the age of 2 (not used in children under 4 years)
- History of flexural involvement (antecubital or popliteal fossa, front of ankles, wrists, or neck)
- History of a generally dry skin in the last 12 months
- Personal history of other atopic disease (in children aged under 4 years, history of atopic disease in a first degree relative may be included)
- Visible flexural dermatitis as per photographic protocol

**Table S1.** Clinical assessment of eczema severity by healthcare professional

	No (%)							
Severity	GP		Nurse		PP		Total	
**Clear**	2	(4)	2	(6)	0	(0)	4	(4)
**Mild**	34	(61)	19	(59)	2	(100)	55	(61)
**Moderate**	17	(30)	11	(34)	0	(0)	28	(31)
**Severe**	3	(5)	0	(0)	0	(0)	3	(3)
**Total**	56	(100)	32	(100)	2	(100)	90	(100)

**Table S2.** Clinical and EASI assessments of eczema severity

	EASI				
Clinical	Clear or nearly clear	Mild	Moderate	Severe or very severe	Total
**Clear**	1	1	0	0	2
**Mild**	17	30	3	0	50
**Moderate**	4	15	4	1	24
**Severe**	0	2	1	0	3
**Total**	22	48	8	1	79

**Table S3.** Clinical and POEM assessments of eczema severity.

	POEM				
Clinical	Clear or nearly clear	Mild	Moderate	Severe or very severe	Total
**Clear**	3	0	1	0	4
**Mild**	3	20	30	2	55
**Moderate**	1	1	18	7	27
**Severe**	0	0	1	2	3
**Total**	7	21	50	11	89

**Table S4.** Most commonly (reported by five or more participants) used emollients

Name of emollient	Number of children prescribed (*n* = 79)
Aveeno^®^	9
Oilatum^®^	8
Cetraben^®^	6
Diprobase^®^	6
Doublebase^®^	5
E45^®^	5
Epaderm^®^	5
Hydromol^®^	5

**Table S5.** Most commonly (reported by two or more participants) used topical **corticosteroid**s

Name of topical corticosteroid	Potency	Number of children prescribed (*n *= 79)
Hydrocortisone 1% (Dioderm, Mildison)	Mild	21
Hydrocortisone 0.5%	Mild	10
Hydrocortisone acetate 1% + fusidic acid 2% (Fucidin H^®^)	Mild	3
Betamethasone valerate 0.1% (Betnovate^®^)	Potent	2
Clobetasone butyrate 0.05% (Eumovate^®^)	Moderate	2
